# Note from the Editor: Additional Changes for Authors

**Published:** 2008-08

Effective 1 August 2008, *EHP* is implementing new page charges of $30.00 per accepted manuscript page. This is a change from our former policy of charging by the printed journal page, and we hope authors will enjoy the benefit of receiving their page charge information earlier in the production process. In calculating page charges, we will include only text pages of the final accepted Microsoft Word version of the manuscript (excluding the first three pages containing the title page, lists of keywords and abbreviations, and outline of section headers).

As of 1 August, we are also instituting a new policy of charging for color figures (e.g., graphs, maps, schematics, photographs). For many years we have been proud to offer our authors free color printing; however, as a government publication, we must find ways to offset our expenses to ensure responsible stewardship of our federal funding. Therefore, authors henceforth will pay $500.00 for the first color figure and $100.00 for each additional figure. (A figure includes, for example, parts A–D, and the complete figure must fit on one page or less.)

Authors will still be billed for page and color charges, but bills will be sent earlier in the production process (soon after acceptance). We are pleased to offer our new Manuscript Central site at http://mc.manuscriptcentral.com/ehp and hope you will enjoy this improved submission system.

